# Family caregiver’s concerns and anxiety about unaccompanied out-of-home activities of persons with cognitive impairment

**DOI:** 10.1186/s12877-023-04025-7

**Published:** 2023-06-28

**Authors:** Shuji Tsuda, Hiroshige Matsumoto, Shun Takehara, Tomoyuki Yabuki, Satoko Hotta

**Affiliations:** 1 Research Team for Human Care, Tokyo Metropolitan Institute of Gerontology, 35-2 Sakaecho, Itabashi, Tokyo, 173-0015 Japan; 2 Department of Community Health Nursing, School of Medicine, The University of Tokyo, 7-3-1 Hongo, Bunkyo, Tokyo, 113-0033 Japan; 3 Department of Occupational Therapy, Faculty of Rehabilitation, Gunma Paz University, 1-7-1 Tonya, Takasaki, Gunma 370-0006 Japan; 4 University of Kochi, 2751-1 Ike, Kochi, Kochi 781-8515 Japan; 5 Graduate School of Health Management, Keio University, Endo, Fujisawa, Kanagawa 4411, 252-0883 Japan

**Keywords:** Anxiety, Overprotection, Family, Caregivers, Out-of-home activities, Cognitive impairment, Dementia

## Abstract

**Background:**

Although people with cognitive impairment highly value social participation in out-of-home activities, their families typically perceive concerns and experience anxiety over such activities. This study aimed to elucidate the underlying concerns and factors associated with family caregivers’ anxiety over the individual’s unaccompanied out-of-home activities.

**Methods:**

In December 2021, we conducted a cross-sectional e-survey of family caregivers of individuals with early-stage cognitive impairment. Caregivers’ concerns about ten common risks related to out-of-home activities were cross-tabulated by specific anxiety levels to examine trend associations. With the variables of caregivers and their individuals across the five domains, we ran logistic regression analyses to determine explanatory models for anxiety.

**Results:**

The study participants were 1,322 family caregivers of people whose cognitive function varied from intact to possible mild dementia according to the Dementia Assessment Sheet for Community-based Integrated Care System 8-item. Significant associations were found between the prevalence of concerns and the degree of anxiety, even without actual experience with the issues of concern. Among the five domains, individual dementia characteristics and social behaviors were the predominant factors attributed to caregiver anxiety. Caregivers’ no anxiety state was significantly associated with: younger age (OR 4.43, 95% CI 1.81–10.81), no detectable cognitive decline (OR 3.34, 95% CI 1.97–5.64), free from long-term care (LTC) (OR 3.52, 95% CI 1.72–7.21), no manifestation of behavioral and psychological symptoms of dementia (BPSD) (OR 13.22, 95% CI 3.06–57.01), and not engaging in unaccompanied out-of-home activities (OR 3.15, 95% CI 1.87–5.31). Their severe anxiety was positively associated with being on LTC (OR 3.39, 95% CI 2.43–4.72) and minor BPSD (OR 1.43, 95% CI 1.05–1.95), and negatively associated with engagement in unaccompanied out-of-home activities (OR 0.31, 95% CI 0.23–0.43).

**Conclusions:**

The study found that family caregivers’ anxiety was associated with concerns about behavioral issues, regardless of actual experiences. There were two significant associations in opposite directions between caregivers’ anxiety and the individual’s engagement in out-of-home activities. In the early phase of cognitive impairment, caregivers may intuitively interpret the individual’s behavior and feel anxious. Educational support may provide reassurance and enable caregivers to facilitate out-of-home activities.

## Background

In caring for persons with dementia, the overarching goal is to help them maintain their quality of life [1]. Social participation in out-of-home activities is recognized as a strategy for enhancing the well-being of persons with dementia, especially in the early phases when self-care functions for domestic life are largely preserved [2, 3]. For these individuals, maintaining the status quo of independent living, comprising both domestic and social autonomy, is of high value [4, 5]. However, the reality of the practical and emotional challenges of living with dementia, such as difficulties with direction or the perceived stigma of a dementia diagnosis, likely translates into a decline in the propensity for out-of-home social engagement [6]. Hackett et al. (2019) found that the percentage of those with cognitive impairment and regular social interactions decreased from 55% pre-diagnosis to 23% post-diagnosis compared to 85% for those with no cognitive impairment [7]. The sharp decline in the maintenance of social interaction from prior-to-onset to pre-diagnosis to post-diagnosis suggests this key strategy of leveraging social participation for enhancing the well-being of persons with dementia faces significant headwinds in its adoption or implementation, and closer attention is warranted towards the barriers and factors surrounding out-of-home activities in the early stages of dementia.

Cognitive impairment in an individual necessitates adjustments in family dynamics when family members take on caregiving responsibilities. As caregivers, family members may feel justified in assuming the oversight of an individual’s independent activity and develop concerns over the perceived risks of such activities [8]. These concerns are predisposed to increase when the activity occurs outside their purview, such as unaccompanied out-of-home social engagement, and may lead to excessive anxiety and overprotectiveness on the part of the caregiver, restricting the attainable independence of the individual and degrading their overall well-being [8–10]. This phenomenon of overprotectiveness is well documented in parents caring for their children and has effects similar to those of caregivers of persons with dementia. Excessive parental anxiety manifests as overprotectiveness to cope with emotional distress and is a critical driver in restricting a child’s age-appropriate autonomy [11, 12]. Similar to overprotecting parents with excessive anxiety, family caregivers of older adults with cognitive impairment may be biased toward overreacting to activities that they perceive as risky and uncertain, which can lead to intense anxiety [9, 11].

One-third of family caregivers of individuals at any dementia stage experience clinically significant anxiety symptoms concerning their caregiving [13]. Considering the prevalence of caregivers’ anxiety increases as the stage of dementia advances [14], most studies have investigated and disentangled the causes and effects of anxiety in caregivers at the later stages of dementia. This type of anxiety is primarily related to care provision, including dealing with behavioral and psychological symptoms of dementia (BPSD) (e.g., agitation, aggression, and psychosis) and the burden of providing physical and practical care [13]. However, the anxiety that early-phase dementia caregivers experience largely differs from that of those in the later phase, as it primarily revolves around the concerns generated by the caregiver’s perception of the risks surrounding the independent activities of the individual, especially when occurring outside of their field of influence or control [8, 15]. This study focused on the anxiety experienced by family caregivers of individuals with early-stage cognitive impairment over unaccompanied out-of-home activities and elucidated the underlying concerns and factors associated with that anxiety.

## Methods

### Study design and ethical procedures

This study analyzed cross-sectional survey data from family caregivers who registered with a commercial marketing research company in Japan. The SFC Research Ethics Committee at Keio University approved all materials and procedures [2021-21]. Written information appeared at the beginning of the e-survey, and electronic consent was obtained from all participants.

### Setting

In December 2021, we conducted an e-survey with Cross Marketing, Inc. They offer a general population panel of approximately 5 million people across Japan and several segmented special panels, including a caregiver panel consisting of family members of older adults in long-term care (LTC).

### Participants

We screened 80,039 persons from the general population panel and 9,325 persons from the caregiver panel. Those who satisfied all the following eligibility criteria were enrolled in the study; (1) adult family caregivers (≥ 20 years old) of individuals (≥ 40 years old) who required attention and support due to possible or definitive dementia, (2) the cognitive status of the individual varied from no detectable cognitive decline to possible mild dementia, as assessed using the Dementia Assessment Sheet for Community-based Integrated Care System 8-item (DASC-8) [16], (3) individuals without a manifestation of disabling BPSD, and (4) family caregivers who lived with or near the individual (within a 30-minute travel distance).

The DASC-8 is an informant rating scale for screening decline in both cognitive function and activities of daily living (ADL), with a total of eight items covering three domains: cognitive function (two items), instrumental ADL (three items), and basic ADL (three items) [16]. The total score ranges from 8 to 32 and classifies cognitive function into three categories: Category (1) Intact cognitive function or possible mild cognitive impairment (score 8–10), Category (2) possible mild cognitive impairment or mild dementia (score 11–16), and Category (3) possible moderate or severe dementia (score 17–32). In consonance with our study objective, we included family caregivers of individuals in Categories 1 and 2 who had some cognitive impairment but whose cognitive functions were supposed to be sufficiently preserved to engage in out-of-home activities alone.

BPSD was assessed by a single question on the individual’s behavior in the last three months with five response options: none, being somewhat anxious or irritated but no assistance needed, expressing severe anxiety and skepticism that requires minor assistance, being extremely distressed and unsettled and needing close supervision, and showing tendencies of self-harm or aggression toward others and needing medical care. Responses were trichotomized, with the first two indicating minimal BPSD, the third indicating minor BPSD, and the last two indicating disabling BPSD. The third category, disabling BPSD, was employed as the exclusion criteria.

### Questionnaire and variables

The questionnaire for the survey was developed by the authors of this study with substantial input from external discussants, including two family caregivers, two healthcare practitioners knowledgeable about dementia care, and one city employee who works for the municipal department of dementia policy.

### Outcome variables: caregiver’s anxiety

The outcome variable was the degree of caregiver anxiety about the individual’s engagement in unaccompanied out-of-home activities, measured by a single question about specific anxiety on a 4-point Likert Scale: very anxious, somewhat anxious, little anxious, and not anxious at all.

### Explanatory variables

To measure the caregivers’ concerns underlying their anxiety, we provided ten items covering frequently cited concerns of caregivers derived from previous research [17], with a four Likert Scale from “very true” to “very untrue.” The answers were dichotomized into two halves to describe their prevalence. For each item of caregiver concern, using a dichotomous option, we also investigated whether the caregivers knew that the object of concern had actually happened to the individual.

To assess the potential explanatory variable for caregivers’ specific anxiety, we devised a five-factor framework. Four were derived from factors reported in a recent systematic review of caregivers’ depression and anxiety: characteristics of the individual with cognitive impairment, caregiver demographics, caregiver psychological and social factors, and dyadic relationships [14]. For the fifth factor, we supplemented the framework with an originally developed factor regarding an individual’s social behaviors in line with the specific object of anxiety.

Individuals’ characteristics included age, sex, living arrangements, cognitive function measured by the DASC-8, development of minor BPSD (applied as a binary variable as disabling BPSD cases were excluded as above), dementia diagnosis, and LTC certification status.

Individuals’ social behaviors included whether they disclose their dementia diagnosis to others, seek help when needed, use mobile phones or smartphones, have friends living with dementia, have friends without dementia, and engage in unaccompanied out-of-home activities. For the out-of-home activities variable, we specified “unaccompanied” activities and provided multiple choices, exemplifying typical out-of-home activities for older adults such as walking, going to libraries and theaters, playing outdoor sports, shopping, and dining out. We aggregated the responses to describe the total number of activities that the individuals with cognitive impairment engaged in and dichotomized the results as no activity or at least one.

Caregivers’ demographics included age, sex, educational status, and perceived economic conditions. Caregivers’ psychological and social factors included perceived health condition, family structure, employment status, experience of volunteering for people with dementia, experience of working in healthcare and welfare services, and social networks measured using the abbreviated Lubben Social Network Scale (LSNS-6). The LSNS-6 scores were dichotomized using validated cutoff points [18]. Dyadic relationship factors included relationships with the individual and whether the caregiver lived with them.

### Statistical analysis

Descriptive statistics were calculated using mean, standard deviation (SD), frequency, and percentage. To elucidate the underlying concerns about anxiety, caregivers’ knowledge of the actual occurrence of the issue and their concerns about it were cross-tabulated to examine the association using the chi-square test. Then, for those with and without knowledge, the prevalence trends of each concern according to the families’ anxiety levels were examined using the Mantel-Haenszel test.

Before exploring the explanatory models, we examined the association between caregivers’ specific anxiety about the individual’s engagement in unaccompanied out-of-home activities and the actual occurrence of these activities. The association formed an inverted U shape, indicating a lower frequency of out-of-home activity in both the upper and lower bounds of the anxiety response spectrum. We trichotomized the responses to (1) very anxious, (2) somewhat anxious and little anxious, and (3) not anxious at all, based on our interpretation that “very anxious” and “not anxious at all” reflect different conditions. The following analyses were conducted using Response 2 as reference. Using logistic regression analyses, we fit the above explanatory variables into two separate explanatory models for caregivers’ anxiety states: very anxious (response 1) and not anxious at all (response 3). For each model, we first ran a bivariate logistic regression and calculated crude odds ratios (ORs), 95% confidence intervals (95% CIs), and p-values for every explanatory variable (crude models). We then performed multivariable logistic regression analyses with selected explanatory variables whose p-values were less than 0.20 in the bivariable analyses (multivariable models). We executed downward stepwise procedures with the threshold p-value of exclusion set at 0.20 to investigate the best explanatory models (final models). Cases with missing data were excluded from analysis. All analyses were computed using IBM SPSS Statistics v. 28.

## Results

We collected responses from 1,600 family caregivers, of whom 1322 satisfied the inclusion criteria and were analyzed. Table 1 shows the descriptive data of the individuals with early-phase cognitive impairment and their caregivers. Regarding individuals’ characteristics, the mean age was 78.2 (SD 11.6); 40.6% were men; 66.7% were classified as having mild cognitive impairment or mild dementia; 49.8% were diagnosed with dementia, and 64.1% were currently engaged in out-of-home activities. The caregivers were 52.4 (SD 13.0) years old, 66.6% were male, more than two-thirds perceived that they had favorable economic and health conditions, 80.0% were currently employed, and 65.5% self-evaluated the size of their social networks as being less than optimal.

**Table 1:** Characteristics of individuals and family caregivers

		n	(%)
Individuals with cognitive impairment			
Age	≤ 64	154	(11.6%)
	65–74	213	(16.1%)
	75–84	514	(38.9%)
	85≤	441	(33.4%)
Sex	Men	537	(40.6%)
Cognitive function by DASC-8	No detectable decline	427	(32.3%)
	MCI/mild dementia	895	(67.7%)
Dementia diagnosis	Diagnosed	658	(49.8%)
Certified for LTC	Certified	608	(46.0%)
BPSD	None	900	(68.1%)
	Minor	422	(31.9%)
Living arrangements	Living alone	177	(13.4%)
Tell their dementia diagnosis to friends & acquaintances	Yes	233	(17.6%)
Seek help when needed	Yes	673	(50.9%)
Use mobile phones or smartphones	Yes	701	(53.0%)
Have friends living with dementia	Yes	114	(8.6%)
Have friends without dementia	Yes	434	(32.8%)
Engage in out-of-home activities alone	Yes	848	(64.1%)
Family caregivers			
Age	20–39	206	(15.6%)
	40–49	309	(23.4%)
	50–59	435	(32.9%)
	60–69	243	(18.4%)
	70≤	129	(9.8%)
Sex	Men	881	(66.6%)
Educational status	College/university	944	(71.4%)
Perceived economic conditions	Good	872	(66.0%)
Perceived health conditions	Good	985	(74.5%)
Size of social networks by LSNS-6	Optimal	456	(34.5%)
Live with own children	Yes	536	(40.5%)
Employment status	Employed	1057	(80.0%)
Experience working in healthcare and welfare services	Yes	156	(11.8%)
Experience volunteering for people with dementia	Yes	77	(5.8%)
Live with the individual	Yes	856	(64.8%)
Relationship to the individual	Spouse	226	(17.1%)
	Child/child-in-law	681	(51.5%)
	Others	415	(31.4%)

DASC-8, Dementia Assessment Sheet for Community-based Integrated Care System 8-item; MCI, mild cognitive impairment; BPSD, behavioral and psychological symptoms of dementia; LTC, Long-Term Care; LSNS-6, Lubben Social Network Scale

### Caregiver’s concerns

Table 2 presents the prevalence of family concerns for the ten common issues that may arise during individuals’ out-of-home activities. When families knew that an individual had experienced an issue, the prevalence of concern ranged from 65.6 to 90.8%. This was significantly higher for every issue than when families did not know whether the individual had experienced the issue. The prevalence of concern ranged from 24.1 to 53.8%.

**Table 2:** Prevalence of family’s concerns by their knowledge of whether the individual experienced the issue

Family concerns	When family did not know whether the individual experienced the issue		When family knew the individual had experienced the issue		p
	n	(%)	n	(%)	
Falling	583	(53.8%)	216	(90.8%)	< 0.001
Being involved in traffic accidents	692	(53.5%)	25	(89.3%)	< 0.001
Missing calls	495	(41.9%)	104	(74.3%)	< 0.001
Bothering neighbors	425	(34.3%)	58	(70.7%)	< 0.001
Getting lost	393	(32.4%)	87	(79.1%)	< 0.001
Having incontinence	371	(29.7%)	59	(79.7%)	< 0.001
Issues of payment	334	(25.6%)	14	(73.7%)	< 0.001
Being insulted by others	329	(25.5%)	21	(65.6%)	< 0.001
Driving	320	(25.0%)	34	(81.0%)	< 0.001
Being accused by neighbors	310	(24.1%)	29	(82.9%)	< 0.001

Computed with chi-square test.

**Fig. 1:**
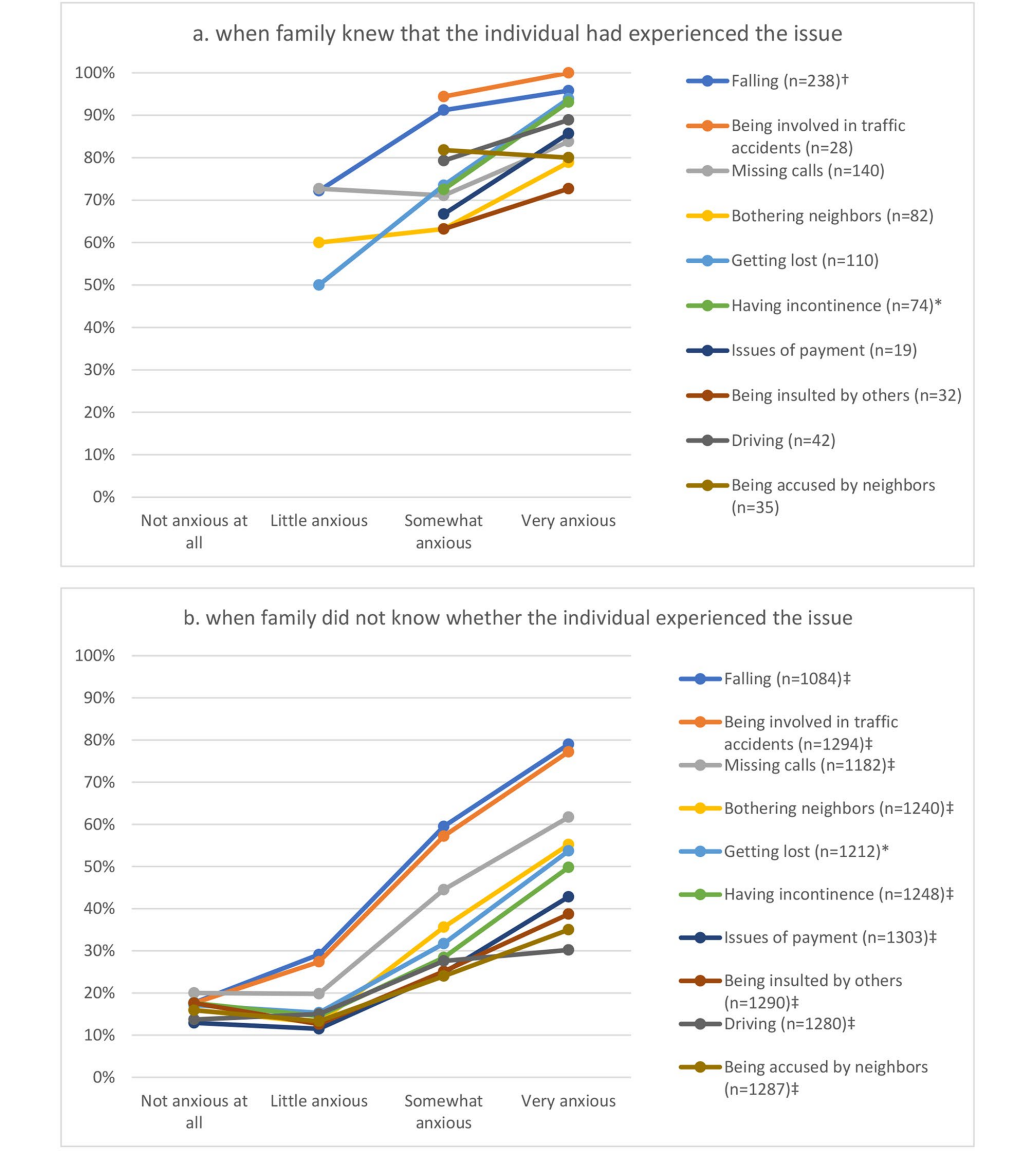
Prevalence of family’s concerns by anxiety levels regarding individual’s out-of-home activities. 1-a. when family knew that the individual had experienced the issue 1-b. when family did not know whether the individual experienced the issue Superscript symbols refer to p-values * <0.05, †<0.01, and ‡<0.001, computed with chi-square test for trend by Mantel-Haenszel method

Figure 1 displays the prevalence of each concern based on anxiety levels. Figure 1a shows that when families knew that the individual had actually experienced a particular issue, 63.2–94.4% of somewhat anxious families and 72.7–100% of very anxious families perceived concerns about that issue. Note that the actual number of families who knew about the individual’s experience was generally low (median 46, range 14–216). Even when families did not know whether an individual had experienced an issue, the prevalence of concern showed a trend association with anxiety levels. Specifically, 30.2–79.0% of very anxious families expressed concerns about the issue (Fig. 1b).

Physical safety was the most prominent source of concern among families, irrespective of their awareness levels. Falls and involvement in traffic accidents were consistently ranked among the top two concerns. Families who were very anxious but lacked awareness of the individual’s actual experiences reported societal inconveniences, such as missing calls and bothering neighbors, as frequent concerns, whereas these issues were ranked much lower at 7th and 9th position by families with similar anxiety levels but with awareness. Among the latter, the 3rd and 4th most common concerns were being lost and experiencing incontinence.

### Associated factors with the anxiety

Family caregivers’ “not anxious at all” state was explained in the final model with 12 explanatory variables, including five from the individual’s characteristics, four from their social behaviors, one from caregiver’s demographics, two from dyadic relationship factors (Hosmer and Lemeshow’s chi-squire 13.1, p-value 0.11) (Table 3). Individual’s younger age ≤ 64 (OR 4.43; 95% CI 1.81–10.81; p-value 0.001), men (OR 1.83; 95% CI 1.10–3.04, p-value 0.020), no detectable cognitive decline by DASC-8 (OR 3.34; 95% CI 1.97–5.64; p-value < 0.001), free from the LTC certification (OR 3.52; 95% CI 1.72–7.21; p-value < 0.001), and no manifestation of BPSD (OR 13.22; 95% CI 3.06–57.01, p-value < 0.001) were the factors significantly associated with a no-anxiety state of caregivers. From the individual’s social behaviors, socially inactive states, such as not engaging in unaccompanied out-of-home activities (OR 3.15; 95% CI 1.87–5.31; p-value < 0.001) was associated with no anxiety. As no use of mobile phones or smartphones was associated with no anxiety (OR 2.35; 95% CI 1.37–4.01; p-value 0.002), the individual’s potentially adaptive coping behaviors did not necessarily show a preferable association with families’ anxiety. From the dyadic relationship factors, the individual’s cohabiting families (OR 1.90; 95% CI 1.01–3.57; p-value 0.047) and spouses in comparison to adult children/children-in-law (OR 2.22; 95% CI 1.06–4.67; p-value 0.035) were more likely to be in a no-anxiety state.

**Table 3:** Final model for caregiver’s no anxiety about individual’s unaccompanied out-of-home activities

		OR	95% CI		p
			Lower	Upper	
Individuals with cognitive impairment					
Age (ref: 85≤)	≤ 64	4.43	1.81	10.81	0.001
	65–74	1.41	0.63	3.15	0.397
	75–84	1.08	0.54	2.19	0.825
Sex	Men	1.83	1.10	3.04	0.020
Cognitive function by DASC-8 (ref: MCI/mild dementia)	Intact	3.34	1.97	5.64	< 0.001
Certified for LTC	Not certified	3.52	1.72	7.21	0.001
BPSD (ref: minor)	Minimal	13.22	3.06	57.01	0.001
Disclose dementia diagnosis to friends & acquaintances	No	2.91	0.93	9.11	0.067
Seek help when needed	No	2.30	1.23	4.30	0.009
Use mobile phones or smartphones	No	2.35	1.37	4.01	0.002
Engage in out-of-home activities alone	No	3.15	1.87	5.31	< 0.001
Family caregivers					
Educational status (ref: College/university)	Junior high/high school	1.51	0.88	2.57	0.133
Live with the individual	Yes	1.90	1.01	3.57	0.047
Relationship to the individual (ref: child/child-in-law)	Spouse	2.22	1.06	4.67	0.035
	Others	1.80	0.96	3.37	0.067

CI, confidence interval; OR, odds ratio; DASC-8, Dementia Assessment Sheet for Community-based Integrated Care System 8-item; MCI, mild cognitive impairment; BPSD, behavioral and psychological symptoms of dementia; LTC, Long-Term Care

Multivariable logistic regression analysis was executed with selected explanatory variables whose p-values were less than 0.20 in each bivariable analysis with all explanatory variables in Table 1. The final model was computed by downward stepwise procedures based on the threshold p-value of exclusion set at 0.20.

The final model for caregivers’ severe anxiety consisted of three variables from the individuals’ characteristics: four from their social behaviors, one from the caregiver’s demographics, and one from the caregiver’s psychological and social factors (Hosmer and Lemeshow’s chi-square 8.30, p-value 0.41) (Table 4). Of these, the variables significantly associated with caregivers’ higher anxiety were being certified for LTC (OR 3.39; 95% CI 2.43–4.72; p-value < 0.001), developing minor BPSD (OR 1.43; 95% CI 1.05–1.95; p-value 0.001), and having friends living with dementia (OR 1.68; 95% CI 1.04–2.69; p-value 0.033). On the other hand, the individual’s engagement in unaccompanied out-of-home activities (OR 0.31; 95% CI 0.23–0.43; p-value < 0.001) and the caregiver’s favorable health conditions (OR 0.60; 95% CI 0.43–0.83; p-value 0.002) were negatively associated with severe anxiety. Regarding the individual’s social behaviors, seeking help when needed was marginally associated with higher anxiety (OR 1.35; 95% CI 0.98–1.83; p-value 0.069).

**Table 4:** Final model for caregiver’s very anxious state about individual’s unaccompanied out-of-home activities

		OR	95% CI		p
			Lower	Upper	
Individuals with cognitive impairment					
Dementia diagnosis	Diagnosed	1.35	0.98	1.87	0.069
Certified for LTC	Certified	3.39	2.43	4.72	< 0.001
BPSD (ref: minimal)	Minor	1.43	1.05	1.95	0.021
Seek help when needed	Yes	1.34	0.98	1.83	0.069
Have friends living with dementia	Yes	1.68	1.04	2.69	0.033
Have friends without dementia	Yes	0.77	0.56	1.07	0.125
Engage in out-of-home activities alone	Yes	0.31	0.23	0.43	< 0.001
Family caregivers					
Perceived health conditions	Good	0.60	0.43	0.83	0.002
Live with the individual	Yes	1.23	0.90	1.68	0.200

CI, confidence interval; OR, odds ratio; BPSD, behavioral and psychological symptoms of dementia; LTC, Long-Term Care

Multivariable logistic regression analysis was executed with selected explanatory variables whose p-values were less than 0.20 in each bivariable analysis with all explanatory variables in Table 1. The final model was computed by downward stepwise procedures based on the threshold p-value of exclusion set at 0.20.

## Discussion

We explored the concerns of family caregivers and the factors underlying their anxiety stemming from the unaccompanied out-of-home activities of individuals with early-stage cognitive impairment. The prevalence of concern was significantly higher when families were aware of the individual experiencing issues than when they were not. Even in families without direct experience, the prevalence of concern increased as anxiety levels increased. The predominant factors of anxiety in caregivers were individuals’ dementia characteristics and social behaviors, where caregivers with the least anxiety cared for individuals who largely maintained their cognitive function but exhibited less active social behaviors. Families with higher anxiety were also associated with the individual’s socially inactive state, specifically with regard to their lack of engagement in unaccompanied out-of-home activities. When individuals employed potentially adaptive coping behaviors, such as using smartphones and seeking help when needed, these activities were unexpectedly associated with higher rather than lower caregiver anxiety.

Even in early-stage cognitive impairment, as the individual develops minor BPSD and increasingly requires more care, the prevalence of anxiety among caregivers regarding unaccompanied out-of-home activities was found to rise. This builds on a body of research that finds greater dementia-related impairment in domestic life is associated with higher anxiety in families and more measures taken to maintain safety [19]. Additionally, our findings that familial concerns without first-hand witnesses or experiences were related to higher anxiety levels indicate that concerns from their intuition could exacerbate anxiety. Substantive safety risks, such as the risk of falling, were the most prevalent concerns among very anxious caregiver respondents without direct experience. These families of individuals who demand closer attention and care may be concerned about the safety risks rationally inferred from their daily activities and take necessary mitigating actions to preserve their safety [20]. By contrast, intuitive concerns about social inconveniences, such as missing calls and bothering neighbors, should not escalate caregivers’ anxiety to the point where they take unwarranted actions.

Our study showed that family caregivers were free from anxiety when individuals were socially inactive (i.e., not engaging in unaccompanied out-of-home activities), although they were cognitively capable. However, when we examined very anxious families, their individuals were socially inactive rather than socially active and required closer attention and care. Interestingly, the same inactive state was related to the opposite responses in families: no anxiety or severe anxiety. This inconsistency may reflect families’ convenient interpretations of the individual’s activities, resulting from their rationalization that their ways of thinking were justified by the circumstances. Non-anxious families may feel less anxious because the individuals lack interest or opportunity for external social interaction. Conversely, families may be very anxious about an individual’s out-of-home activities because they depend on care, are uncertain about their behaviors and capabilities, and do not regularly engage in unaccompanied out-of-home activities [20]. Family caregivers in this study tended to view the individual’s adaptive coping efforts, such as utilizing smartphones and seeking help when necessary negatively, which supports the inference that families may exercise a convenient interpretation of the individual’s condition and behaviors.

Caregivers who misinterpret an individual’s behaviors and compound anxiety may resort to potentially suboptimal countermeasures such as surveillance and restrictions [21]. The balance between the caregiver’s mitigation of risks and the individual’s social autonomy is the primary challenge that the family faces in the early stages of cognitive impairment and should be treated with appropriate care and deliberation [22]. Families inexperienced in caring for persons with cognitive impairment should seek available resources, instructions, or counseling to educate themselves on their condition and caregiving skills.

Our findings suggest a clear need for educational and emotional support resources for caregivers to alleviate excessive anxiety over their individual’s autonomous activities and to avoid misinterpretation of their efforts to cope with and accommodate their condition. At present, family caregivers though well intentioned, are not well supported in obtaining the knowledge and skills required to address the risk-inherent autonomous activities of people with dementia [23]. Especially in the early phase of cognitive impairment, when this new paradigm imposes adjustments and changes to family relationships and responsibilities, family caregivers need to be instructed on how to balance their internal concerns for the safety of the individual, while preserving their social autonomy and overall well-being [22, 24]. Enhancing support resources at an individual level will further complement social-level strategies such as Dementia Friendly Communities, which are designed to provide safe and inclusive communities for people with dementia to participate in and contribute to, where family members can have peace of mind [25].

This study has some methodological limitations. This study employed an e-survey conducted by a commercial marketing company that posed sampling bias issues. The study participants may not be fully representative, as male-dominant caregivers and only 17% of spousal caregivers were found in the baseline characteristics. Those who are more comfortable with technology may be over-represented. The outcome was measured using a single question, which may not fully capture the different dimensions of emotion. We deliberately chose this measurement method in line with the study purpose of delineating the specific anxiety of caregivers. The anxiety question seemed to work as expected, referring to the consistent trend associations between the prevalence of concerns and degree of anxiety confirmed in Table 2. Although a cross-sectional study design can reveal an association between variables, it cannot establish causality. The observed associations may also be subject to reverse causality or unmeasured confounding. As such, the results of this study should be interpreted with caution. Further studies using longitudinal or experimental designs may be needed to confirm the direction of causality and to better understand the underlying mechanisms of the observed association.

We also note the potential influence of the COVID-19 pandemic. Older adults with cognitive impairment are susceptible to infection due to the difficulty with following safeguarding procedures, and they are vulnerable once infected [26]. These individuals may have reduced their engagement in out-of-home activities to comply with social restrictions and lower their risk of infection. On the other hand, the caregivers’ anxiety may have flared up and reflected the prevalence of severe anxiety in this study, which was conducted during the pandemic.

## Conclusions

Family caregivers of individuals with early-stage cognitive impairment experience higher anxiety because they are concerned about the individual’s behavioral issues. This is true even without direct experience with the issues. While families are generally free from anxiety about an individual’s unaccompanied out-of-home activities when they are socially inactive yet capable, they become very anxious when individuals are socially inactive and require more care. In particular, in the early phase of cognitive impairment, when inexperienced families may apply biased interpretations of an individual’s behaviors, they should seek available educational resources on the condition and caregiving skills. The instructions should emphasize how to interpret an individual’s condition and behaviors, including adaptive coping strategies and behavioral issues that may raise concerns. This will help caregivers balance their anxiety about individual safety by preserving their social autonomy and overall well-being.

## Data Availability

The dataset used and analyzed in this study is available from the corresponding author upon reasonable request.
